# Evaluation of the effect of targeted Mass Drug Administration and Reactive Case Detection on malaria transmission and elimination in Eastern Hararghe zone, Oromia, Ethiopia: a cluster randomized control trial

**DOI:** 10.1186/s13063-022-06199-8

**Published:** 2022-04-07

**Authors:** Semira Abdelmenan, Hiwot Teka, Jimee Hwang, Samuel Girma, Sheleme Chibsa, Eric Tongren, Matthew Murphy, Mebrahatom Haile, Dereje Dillu, Jawar Kassim, Sinknesh Behaksra, Fitsum G. Tadesse, Joshua Yukich, Yemane Berhane, Alemayehu Worku, Joseph Keating, Ayele Zewde, Endalamaw Gadisa

**Affiliations:** 1 Addis Continental Institute of Public Health, Addis Ababa, Ethiopia; 2 U.S. President’s Malaria Initiative, Addis Ababa, Ethiopia; 3 U.S. Centers for Disease Control and Prevention, Atlanta, GA USA; 4 U.S. Centers for Disease Control and Prevention, Addis Ababa, Ethiopia; 5 Ministry of Health, Addis Ababa, Ethiopia; 6 Oromia Regional Health Bureau, Addis Ababa, Ethiopia; 7 Armauer Hansen Research Institute, Addis Ababa, Ethiopia; 8 Tulane University, New Orleans, LA USA

**Keywords:** Malaria, Ethiopia, Reactive case detection, Targeted mass drug administration, Cluster randomized controlled trial

## Abstract

**Background:**

Reactive and proactive case detection measures are widely implemented by national malaria elimination programs globally. Ethiopia decided to include Reactive Case Detection (RCD) and targeted Mass Drug Administration (tMDA) approaches as part of their elimination strategy along with rigorous evaluation. The purpose of this study is to compare the impact of RCD and tMDA on malaria elimination over the 2-year study period, by looking at the annual parasite incidence before and after the intervention.

**Methods:**

The study will be conducted in the East Hararghe zone of Ethiopia. Malaria transmission in the area is low to moderate. This study will deploy a community-based, three-arm, cluster-randomized control trial implemented over 2 years. Forty-eight clusters (16 clusters per arm) will be selected based on the annual number of confirmed malaria cases seen in the cluster. All clusters will receive the current standard of care in terms of malaria elimination interventions provided by the national malaria control program. In addition, following the identification of malaria parasite infection, individuals who reside within a 100-m radius of the index case will receive a diagnosis for malaria and treatment if positive in the RCD arm or presumptive treatment in the tMDA arm. The primary effectiveness endpoint will be measured at baseline and endline for each intervention arm and compared to the control arm using a difference in difference approach.

**Discussion:**

This randomized controlled trial will provide evidence of the impact of the proposed intervention approaches for malaria elimination.

**Trial registration:**

ClinicalTrials.gov NCT04241705. Registration date: January 27, 2020.

## Administrative information

Note: the numbers in curly brackets in this protocol refer to SPIRIT checklist item numbers. The order of the items has been modified to group similar items (see http://www.equator-network.org/reporting-guidelines/spirit-2013-statement-defining-standard-protocol-items-for-clinical-trials/).

**Table:** 

Title {1}	Evaluation of the effect of targeted Mass Drug Administration and Reactive Case Detection on malaria transmission and elimination in Eastern Hararghe zone, Oromia, Ethiopia: a cluster randomized control trial study protocol
Trial registration {2a and 2b}.	clinicaltrials.govIdentifier: NCT04241705
Protocol version {3}	December 18, 2019 Version 2.3
Funding {4}	U.S. President’s Malaria Initiative is funding the study.
Author details {5a}	1.Addis Continental Institute of Public Health, Addis Ababa, Ethiopia 2.U.S. President’s Malaria Initiative/ Addis Ababa, Ethiopia 3.U.S. Centers for Disease Control and Prevention, Atlanta, GA and Addis Ababa, Ethiopia 4.Ministry of Health, Addis Ababa, Ethiopia 5.Oromia Regional Health Bureau 6.Armauer Hansen Research Institute, Addis Ababa, Ethiopia 7.Tulane University, New Orleans, LA
Name and contact information for the trial sponsor {5b}	Endalamaw Gadisa, Armauer Hansen Research Institute, Addis Ababa, Ethiopia Email address: endalamaw.gadisa@ahri.gov.et
Role of sponsor {5c}	The study sponsor and funders had role in the design of this study, its execution, and decision to submit results.

## Introduction

### Background and rationale {6a}

Globally, the past decade has witnessed substantial declines in malaria morbidity and mortality. Despite this, malaria claimed over 435,000 lives in 2017; most of whom were African children. Progress towards elimination in specific areas, however, appears to be slowing down in recent years; the 2018 global estimate of malaria incidence (228 million cases) was 9 million higher than the previous year [1].

Ethiopia has a unique malaria eco-epidemiology based on altitude. The predominant malaria species, *Plasmodium falciparum* (*p. falciparum,* ~ 60%) and *Plasmodium vivax* (*p. vivax*, ~ 40%), co-exist with *Plasmodium malariae* and *Plasmodium ovale* infections (< 1%) [2]. Malaria transmission is seasonal and unstable. Sixty percent of the population is currently at risk of malaria infection (3). Scaling up interventions such as long-lasting insecticidal nets (LLINs), indoor residual spraying (IRS), and access to quality diagnosis and treatment in affected populations over the last decade resulted in significant annual malaria incidence reductions between 2011 and 2016. During the same period, the annual incidence of malaria-associated death was reduced from 2.1 to 1.1 deaths per 100,000 [2]. Similarly, a study using global burden of disease data showed a decline; a reduction of malaria morbidity and mortality by more than 88% and 96.5%, respectively, in the last 25 years [4]. Furthermore, the malaria transmission map has been shrinking with the number of districts classified as high transmission declining from 138 in 2013 to 54 in 2016 [3].

Encouraged by the observed reductions, the Ministry of Health (MOH) of Ethiopia set an ambitious goal to be malaria-free by 2030. To achieve this goal, it has taken on a stepwise strategy to eliminate malaria at the district level first, starting in 239 low transmission districts [3].

To optimize case management in the context of malaria elimination, the national treatment guideline was amended to include single low-dose primaquine (PQ) (0.25 mg/kg) in addition to artemether-lumefantrine (AL) for all passively detected *P. falciparum* cases, to use PQ (0.25 mg/kg) for 14 days in addition to chloroquine (CQ) for *P. vivax* infections in all transmission settings as recommended by WHO [5]. Both AL and CQ are schizonticidal drugs that treat blood-stage infections. PQ at differing doses can treat both *P. falciparum* gametocytes, which is responsible for onward transmission, as well the *P. vivax* hypnozoites responsible for future relapsing episodes.

In many countries, the utilization of PQ is hindered by the widespread prevalence of glucose-6-phosphate dehydrogenase (G6PD) deficiency, which is common in malaria-endemic areas [6, 7]. PQ administration in G6PD deficient patients is associated with dose-dependent hemolysis after treatment. The very low prevalence or absence of G6PD deficiency in Ethiopia prompted the approval of PQ administration without G6PD testing, with close monitoring of patients [5]. Furthermore, a recent review summarizing the safety of six decades of PQ use in approximately 200 million people found only 14 deaths associated with G6PD deficiency [8].

In addition to optimizing case management, case investigation and response strategies (active surveillance) such as reactive case detection (RCD) and targeted mass drug administration (tMDA) of passively detected cases are being considered as part of the elimination strategy. Cautious of the limited evidence that tMDA or RCD works in elimination settings, there is a need to optimize both the operational implementation of such strategies and the information systems used so as to allow for testing the effect of these strategies on malaria outcomes in elimination settings.

In settings like Ethiopia, having multiple parasite species circulating will certainly have implications for malaria elimination. There is already global evidence that treatment failure, recurrence of *P. vivax*, and mixed infection whereby the treatment of *P. falciparum* results in *P. vivax* relapse, should be considered when developing elimination strategies [9–11]. The inclusion of 14 days of PQ for radical cure (clearing both blood and liver stage infections) of *P. vivax* and gametocidal treatment for *P. falciparum* of all patients with malaria in regions of co-endemicity could therefore have important implications for ensuring the removal of parasites.

Under low transmission settings, acquired immunity and low parasite clonal complexity may allow most infected hosts to remain asymptomatic with undetected, low-density parasitemia [12, 13]. As a result, the proportion of infections detected by standard diagnostics, microscopy and rapid diagnostic test (RDT), is significantly lower in these settings [14, 15]. Several studies showed large reservoirs of asymptomatic carriage [16–18] in Ethiopia. Although less likely than higher-density, symptomatic infection, low-density asymptomatic carriage has been shown to contribute substantially to mosquito infection, and thus onward transmission of malaria [15, 19, 20]. Furthermore, infections that are commonly termed “asymptomatic” might have clinical consequences for the individual [21, 22]. Despite concerns that treating asymptomatic infections may lower immunity, treatment of these infections did not appear to increase the chance of re-infection in the subsequent season [23].

The highest probability of finding additional cases surrounding the index case was noted to be in the immediate household and within a 100- to 200-m radius of the index case [24, 25]. Based on these factors, reactive and proactive case detection measures are widely implemented by national malaria elimination programs globally. Similarly, the Ethiopian MOH decided to include RCD and tMDA approaches as part of the elimination strategy along with rigorous evaluation. The purpose of this study is to compare the impact of RCD and tMDA on malaria elimination over the 2-year study period, by looking at the annual parasite incidence before and after intervention.

### Objectives {7}

The primary objective of this study is to measure the effect of RCD and tMDA on malaria annual parasite incidence (API). Secondary objectives include measuring the prevalence of malaria, as well as the measurement of intervention coverage, acceptability of interventions, antimalarial treatment adherence, serious adverse events, cost and cost-effectiveness of the RCD and tMDA strategies relative to a control arm.

### Trial design {8}

**Fig. 1:**
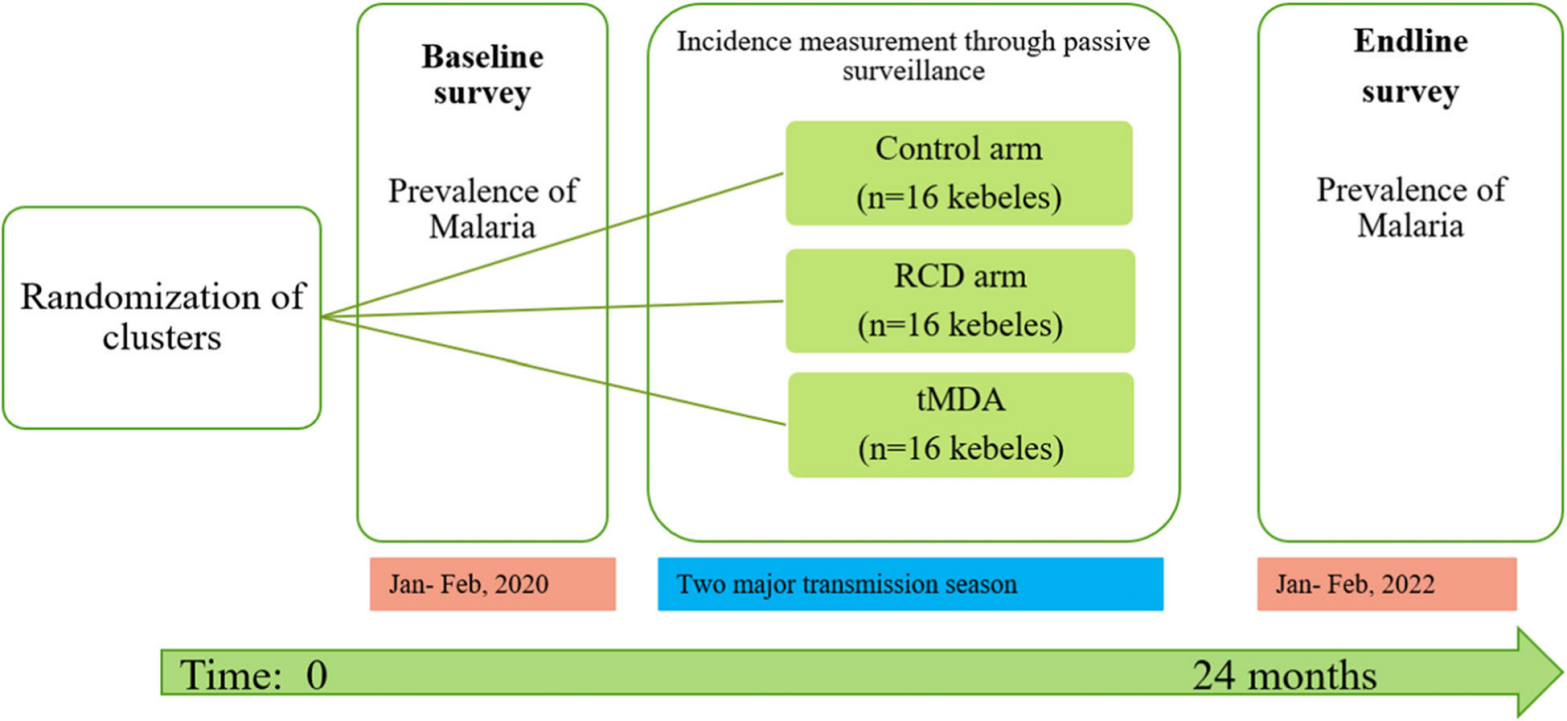
Randomization procedure, interventions, and evaluation timeline

This study will deploy a three-arm, cluster-randomized control trial implemented over 2 years. Kebeles (the smallest administrative unit with 5000 estimated residents) will be the unit of randomization (referred to as clusters hereafter). A cluster has one Health Post (HP) linked to the nearest Health Center (HC) providing health services in the catchment area. Forty-eight clusters (16 clusters per arm) will be selected based on the annual number of confirmed malaria cases seen in the cluster. Those clusters which reported confirmed malaria cases between 1 and 50 will be eligible for the study (Fig. 1).

## Methods: participants, interventions, and outcomes

### Study setting {9}

The study will be conducted in the East Hararghe zone of Ethiopia. The zone has 20 woredas (an administrative unit equivalent to a district), 4 town administrations, and 587 kebeles (the lowest administrative unit) out of which the majority, 541, are classified as rural. The estimated population is 3,737,469, of which approximately 48% are female [26]. This population is served by 548 HP, 115 HCs, 5 hospitals, and 258 private health facilities. A HP serves ~ 5000 people, while a HC has a catchment population of ~ 25,000; HPs report to their respective HC.

**Fig. 2:**
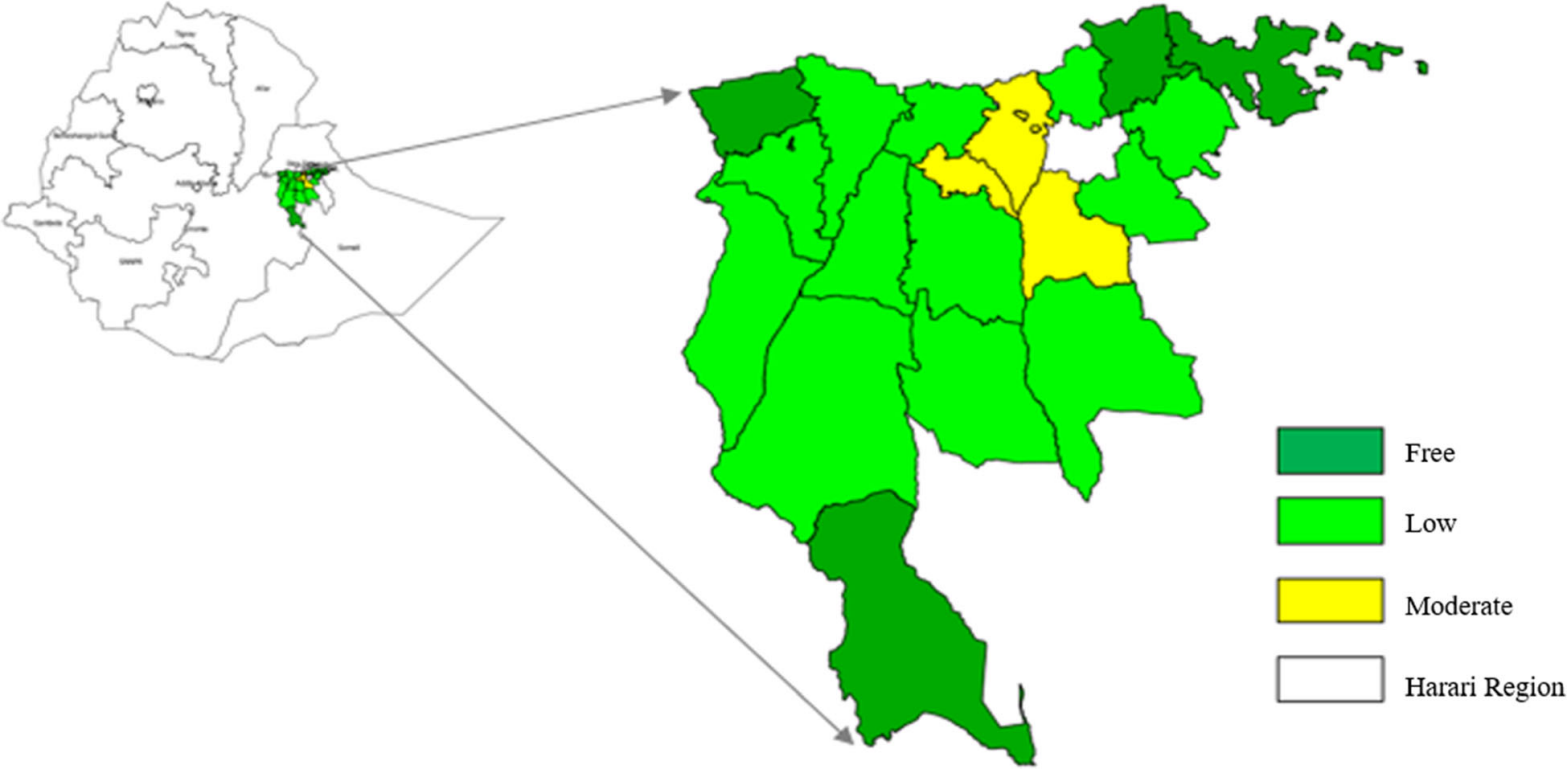
Malaria transmission settings of woredas in East Hararghe zone, Oromia regional state, Ethiopia, 2017

Malaria transmission in the study area is low to moderate. According to the MOH updated malaria risk stratification in 2017, 5 woredas were stratified as malaria-free, 13 woredas are categorized as low (API < 5), and 6 woredas are classified as having moderate transmission (API 5 to 100/1000 population) (see Fig. 2 ). There are no woredas in the high malaria stratum (API ≥100/1000 population) in the study area. In 2017, the East Hararghe zone tested 58,690 suspected cases and 15,245 of them were positive for malaria. Of those 13,179 (86%) were *P. falciparum* cases, 2066 (14%) were *P. vivax* cases, and treated according to the national treatment guidelines. In addition,1669 (9.9%) clinical cases were treated without parasitological confirmation, which makes the average API 4.5/1000 population. In addition to increased treatment, the zone has also rolled out other prevention interventions such as LLINs, IRS, and a relatively strong surveillance system.

### Eligibility criteria {10}

Inclusion and exclusion criteria will be applied at the community and individual levels. The malaria-free woredas will be excluded from the study; of the remaining 19 woredas with ongoing malaria transmission, 10 with the highest API in 2018 will be eligible for the study. Kebeles in the eligible woredas with ongoing PMI supported malaria surveillance and targeted for elimination with comparable malaria control interventions in place, plus annual confirmed malaria cases between 1 and 50 will be eligible for inclusion. Lastly, all residents within the eligible kebeles will be eligible for inclusion.

Exclusion criteria for individuals are (1) children less than 6 months of age or < 5 kg; (2) known allergy or history of adverse reaction or chronic/congenital disease contra indicated to any of the intervention drugs; (3) individuals with severe malnutrition or signs of severe disease, with evidence of any organ failure or hemoglobin (Hgb) level < 8gm/dl; and (4) household members already covered by the intervention drugs less than or equal to one month before the intervention. In addition, the following individuals will be excluded from receiving primaquine: (1) individuals with G6PD level ≤4 U/Hgb, (2) pregnant women, and (3) lactating women breastfeeding infants less than 6 months of age or with unknown G6PD status.

All intervention activities will be conducted by trained health care and study team members. Those study staffs participating in intervention activities will be trained in informed consent procedures, administration of pharmaceuticals, and data collection involving both survey data and blood specimens.

### Who will take informed consent? {26a}

Informed consent will be obtained from all study participants in all arms of the study. The informed consent process and documentation will be administered from trained study team members at the time of data collection at the household or health facility where the subject was identified.

### Additional consent provisions for collection and use of participant data and biological specimens {26b}

As part of the consent process, the informed consent form will also describe the potential use of the collected data for secondary analyses. Any request for secondary analyses needs approval from the institutional review board (IRB) of the requesting institute. Data will be aggregated and no personal identifying information will be distributed as part of any secondary analyses.

## Interventions

### Explanation for the choice of comparators {6b}

This study will have three arms: a control arm, RCD arm, and tMDA arm. Kebeles will be randomized to one of these arms. As all kebeles in the study area are comparable on key characteristics, it is likely that randomization will produce similar comparators, with potential confounders distributed evenly throughout control and intervention areas.

Kebeles in the control arm will receive the current standard of care in terms of malaria elimination interventions provided by the national malaria elimination program, which are referred to as “optimized malaria control interventions” defined as strengthened surveillance and commodities management systems, scale-up of vector control and case management services, and social and behavior change communication to seek prompt treatment and use LLINs. Case management will follow national treatment guidelines. This includes passive detection of malaria cases and treatment with AL plus single low dose PQ (0.25 mg/kg once) for *P. falciparum* cases; CQ plus 14 days of PQ (0.25 mg/kg daily) for *P. vivax* cases; and AL plus 14 days of PQ (0.25 mg/kg daily) for mixed infections as well as follow-up at a health facility on days 3, 7, and 13 for those receiving 14 days of PQ to assess adverse events and adherence as per the national treatment guidelines.

### Intervention description {11a}

Kebeles in the RCD arm will get optimized malaria control interventions as described above. In addition, following identification of parasite infection via microscopy or RDT among passively-detected index cases at the HP or HC, individuals who reside within a 100-m radius of the index case will receive diagnosis for malaria using a conventional RDT. Positive individuals will receive treatment and follow-up as per the national treatment guidelines. Fourteen days of PQ will only be administered to those found to have G6PD levels ≥4 U/Hgb on the standard G6PD test (SD Biosensor, South Korea). Additional procedures will include the collection of a dried blood spot (DBS) for molecular confirmation and secondary analyses.

Kebeles randomized to the tMDA arm will get the optimized malaria control interventions as described above. In addition, following the identification of a parasite infection via microscopy or RDT among positive index cases at the HP or HC, individuals who reside within a 100-m radius of the index case will receive presumptive treatment with AL plus 14 days of PQ (0.25 mg/kg daily); 14 days of PQ will only be administered to those found to have G6PD level ≥4 U/Hgb.

### Criteria for discontinuing or modifying allocated interventions {11b}

A patient who developed a serious adverse event will be referred to the nearby hospital and the study will facilitate the transportation. If any of the following symptoms occur, the patient will be withdrawn from the study and will be managed at the HC or referred to a nearby hospital for better care:

- Dark urine with Hillmen [27] urine color score of greater than 5
- Hypersensitivity reaction necessitating the discontinuation of the study drug(s), as indicated above
- Symptomatic anemia or hemoglobin level < 5 g/dl

### Strategies to improve adherence to interventions {11c}

In both RCD and tMDA arms, one family member will be recruited as a treatment supporter and will be instructed to directly observe pill(s) being swallowed at all times.

### Relevant concomitant care permitted or prohibited during the trial {11d}

Subjects who do not attend the clinic for follow-up will be traced by the study team member who will counsel the subject on the importance of follow-up care. Any subject in the intervention arms who develop a serious adverse event (SAE) will be referred to a hospital and the study will facilitate transportation.

### Provisions for post-trial care {30}

None, beyond the standard care of patients in the Ethiopian malaria treatment guideline. Study participants will exit the study after the completion of the intervention. There will be no further follow-up.

### Outcomes {12}

#### Primary endpoint

The effect of RCD or tMDA will be defined as a change in malaria API among kebele residents measured by microscopy or RDT tests at HCs or HPs. The malaria API will be defined as all passively confirmed malaria cases over a period of 12 months among residents of the selected kebele divided by the estimated population in the selected kebele multiplied by 1000. The primary effectiveness endpoint will be measured at baseline, and at endline for each intervention arm and compared to the control arm using a difference in difference approach.

#### Secondary Endpoints

- Prevalence of malaria infection, measured cross-sectionally at baseline and 24 months later at endline using a household survey in all three arms: prevalence will be estimated using RDT and molecular testing.
- Proportion of households reporting intervention coverage
- Proportion of household respondents reporting acceptability of intervention in RCD and tMDA arms, measured cross-sectionally at endline using a household survey in all three arms
- Proportion of household respondents reporting adherence to antimalarials
- The total costs of RCD and tMDA, measured using program records
- Cost-effectiveness of intervention (RCD and tMDA) relative to control arm standard of care measured using program records and financial reports
- The proportion of imported versus locally acquired incident cases, measured cross-sectionally at baseline and at endline using a household survey with travel questions in all three arms, in addition to measurement at health facilities during visits
- Malaria burden as measured by antigen, serology, and molecular testing
- The proportion of adverse events and serious adverse events identified in the RCD and tMDA arm

### Participant timeline {13}

The study duration is 24 months. Subjects residing in selected study arms will receive a baseline household survey at time zero, and a second after 24 months. Routine health facility visits will take place as usual throughout the study period.

### Sample size {14}

Assuming that the RCD intervention would decrease API by at least 40% and the tMDA intervention by more than that, based on the previous year’s data, we assume a baseline API of 7.8/1000 population and expect to decrease to 4.7/1000 population in the intervention arms. Sampling 16 clusters in each group or arm with an average of 500 person-years per cluster achieves 80% power to detect a difference of − 0.03100 between the treatment event rate 0.047 and the control event rate 0.078. The between-cluster coefficient of variation in the control group and in the intervention group was assumed to be 0.5. A two-sided *t* test of the event-rate difference was used with a significance level of 0.05.

Total population of a kebele is estimated to be 5000 people; data from the HP was used to determine the burden to inform sample size calculation. The cross-sectional survey population will include all residents in 20 randomly selected households in 48 study clusters (16 per arm). A total of 960 households will be needed to detect a reduction in sero-prevalence from 17 to 8% between baseline and endline between arms (assuming an intra-cluster correlation of 0.005, a confidence level of α=0.05, and a power of > 80%).

### Recruitment {15}

Random selection will be used to recruit respondents for the household survey and prevalence testing. Routine health facility visits will be used to recruit index cases for the intervention strategies, and for the collection of incidence data.

## Assignment of interventions: allocation

### Sequence generation {16a}

N/A

### Concealment mechanism {16b}

N/A

### Implementation {16c}

Intervention and standard of care activities in all arms will be implemented by the national malaria elimination program and study team members.

In the control arm, the surveillance officer based at the district level will monitor the collection of malaria data by health staff at HPs/HCs using standard reporting form(s), which includes a follow-up and adherence assessment for primaquine treated cases. In addition, the officer will document any other interventions not routinely conducted in the cluster. In short, the surveillance officer will ensure that the standard of care is executed in all the control kebeles by closely working with the woreda focal persons and other responsible government institutes.

In the study clusters, individuals with a history of fever or axillary temperature ≥ 37.5 °C and who seek care at a HC or HP will be tested with microscopy or RDT, respectively, per national guidelines [28]. Individuals testing positive will receive antimalarial treatment with AL+ a single dose of PQ for *P. falciparum*, CQ+ 14 days of PQ for *P. vivax*, or AL + 14 days of PQ for mixed infections. Women between 12 and 49 years of age with an unknown pregnancy status will be tested for pregnancy as per national guidelines, upon obtaining verbal consent. For women ages 12–17 years, verbal consent from parents and verbal assent from the mature minor will be obtained for a pregnancy test. Primaquine will not be administered to pregnant women or lactating women who are breastfeeding infants less than 6 months of age. Women in the first trimester of pregnancy will be treated with quinine tablets for *P. falciparum*, while those in the second and third trimesters will receive AL for *P. falciparum* or mixed case. CQ will be given for *P. vivax* cases in all trimesters. Age-eligible women refusing the pregnancy test will only receive appropriate first-line blood-stage treatment without PQ as per the national treatment guideline. Infants < 6 months of age will be treated with standard treatment according to national guidelines, which does not include PQ.

In the RCD arm, the surveillance assistant will notify the district malaria focal person and the study surveillance officer within one day of a positive diagnosis at the HP or HC. Case investigation will be done within three days using the case investigation form at their respective facilities. Within 7 days, the study team in charge of RCD, surveillance officer, surveillance assistant, and health extension workers (HEWs) will visit the patient at her/his home. Then, all consenting index household members and members of households within a 100-m radius will be tested for malaria using RDT. In addition, three dried blood spots will be collected on Whatman 903 filter paper for subsequent laboratory-based malaria testing. Individuals found positive by RDT will be treated with AL plus a single dose of PQ for *P. falciparum*, CQ plus14 days of PQ for *P. vivax*, or AL plus 14 days of PQ for mixed infections. G6PD testing will be done in *P. vivax* and mixed infections using the SD Biosensor STANDARD G6PD test. The result will be provided to the participant and 14 days of primaquine administered only to those confirmed to have a G6PD value ≥4 U/Hgb. A designated family member will serve as a treatment supporter and directly observe the medicines being taken every day. Women of childbearing age with an unknown pregnancy status will be tested for pregnancy and provided treatment as per the national treatment guidelines. Infants < 6 months of age will be excluded from the study.

In the tMDA arm, case reporting and investigation will be done as stated in the RCD arm. The tMDA team will visit the diagnosed index case, at his/her home within 7 days. All consenting and eligible household members and members of households within a 100-m radius will be treated without RDT testing for malaria with AL plus 14 days of PQ after receiving G6PD testing and only those with G6PD value ≥4 U/Hgb will be given PQ. The G6PD test results will be provided to the participant. Febrile household members will be tested with RDT and will be treated regardless of the test result. If the RDT is negative, they will be advised to seek further care at the nearest health facility. Women of childbearing age with an unknown pregnancy status will be tested for pregnancy with consent. Women in the first trimester of pregnancy will be treated with quinine while those in the second and third trimesters will receive AL as per the national guidelines. A designated family member will serve as a treatment supporter and directly observe the medicines being taken every day. Age-eligible women refusing the pregnancy test will be excluded from the study. Infants < 6 months of age will be excluded from the study.

### Implementation monitoring

To ensure participants’ adherence to their medication, the community will be educated on how to take their medications, the side effects of the treatment, and the importance of adherence. Participants in the control arm, will follow the national guidelines which requires follow-up on days 3, 7, and 13 using the national follow-up form if taking 14 days of PQ. Those receiving other regimens will only return to the health facility if presenting with symptoms as per the national guidelines. A more detailed adverse events assessment will be conducted at all follow-up visits. Similarly, for participants in the RCD and tMDA arms participants taking 14 days of PQ will be instructed to come to the health post on days three, seven, and thirteen after the initial treatment to assess adherence to and adverse events of treatment. In the RCD arm, those testing positive for *P. falciparum* and administered only a single low dose PQ will be asked to return for follow-up only on day 3 to assess for adherence and adverse events. During the follow-up, the surveillance assistant will administer a brief questionnaire regarding adherence to the treatment regimen and ask about any side effects experienced. He/she will also conduct a pill count and check for any leftover medications or empty drug packaging. In addition, if a participant fails to come to HP/HC for follow-up on scheduled dates, the surveillance assistant will follow up with a phone call and/or a home visit. During the home visit, there will be a questionnaire to determine the reason for missing the follow-up visit.

### Laboratory tests and quality assurance

According to the Ethiopian national malaria treatment guideline, laboratory confirmation of malaria is done using RDT at the HP level and microscopy at HC’s and above. G6PD test is conducted by SD Biosensor standard G6PD test. To ensure quality laboratory diagnosis, the study team at the initiation of the study will provide appropriate training to the laboratory personnel at the HC, and the HEWs from the intervention kebeles. The study team will make regular supervision visits to the study sites and perform quality assessments using the procedure recommended by the national malaria diagnostic quality assurance scheme. The project will also make sure all laboratory consumables will be quality checked prior to importation and utilization at the sites. An internal quality check will also be instituted on a subsample of specimens. The following tests will be performed to strengthen the interpretation of the findings:

#### Nucleic acid extraction and quantitative PCR

DNA will be extracted from 6 mm diameter punches that will be treated with 20 μl Proteinase K (QIAGEN) in a total volume of 200 μl containing Tissue Lysis Buffer (QIAGEN) at 56 °C overnight in a water-bath, followed by extraction using commercial DNA extraction kit (QIAGEN). Real-time quantitative (qPCR) for parasite detection will be performed by targeting the 18S small rRNA gene for Pf and Pv using primer and probe sequences as described [29, 30]. *P. falciparum* parasites will be quantified using standard curves generated from a serial dilution of NF54 ring-stage parasites [29]. *P. vivax* parasite quantification will be done using plasmid constructs to infer copy numbers as described before. Blood samples in RNA protect buffers will be used for extraction of RNA using the RNeasy Mini Kit (QIAGEN) for gametocyte quantification, gametocyte commitment and maturation assays, sex ratio estimation [31], asexual stage parasites detection, and expression level of regulators of the balance between reproduction and replication [32–34].

#### Serology

Sero-prevalence will be determined using LUMINEX-based multiplex assays as described before [35]. Age-specific sero-conversion and sero-reversion rates over the 2 years will be used to monitor changes in transmission and malaria exposure over time. Absence of antimalarial antibodies, particularly in children, will show the success of tMDA or RCD interventions. Antigen selection will be informed by recent studies and a panel of antibodies characterized to indicate a recent change in transmission will be included [36].

#### Antigen test

Testing for pan-Plasmodium antigens aldolase and LDH will determine active infection with malaria parasite and if the infecting species is *P. falciparum*, by the presence of HRP2 antigen. Any samples found to be positive for any antigen will have total DNA extracted from DBS samples for malaria-specific PCR reactions. As the multiplex laboratory antigen test is more sensitive than field RDTs, estimates can be generated for how well field RDTs performed in detecting malaria antigens.

Additional molecular work to assess parasite relatedness, genetic variants of G6PD, and drug metabolism, e.g., cytochrome p450 2D6 as it relates to malaria as well as antigen and antibody testing for diseases of public health importance may be conducted. No other human genetic testing including whole genome sequencing or HIV testing will be undertaken.

## Assignment of interventions: Blinding

### Who will be blinded {17a}

It was not possible to blind both the participants and health professionals. This is an open-label trial.

### Procedure for unblinding if needed {17b}

Not applicable. There is no blinding as part of the interventions in this study.

## Data collection and management

### Plans for assessment and collection of outcomes {18a}

This study will deploy both secondary and primary data collection methods. The secondary data will be used to collect community-level data captured by the routine health systems, while primary data collection will be used to collect household-level information using household surveys.

#### Routine data

Five years of retrospective malaria case data will be obtained from the routine health management information system (HMIS), population census data, and meteorological data will be obtained from districts and the National Meteorological Agency. These data will be used to describe the study settings using descriptive statistics, mean values for rainfall and temperature, and API (annual incidence = number of malaria cases/1000 target population each year).

#### Survey-based data

There will be baseline and endline survey. The survey will be conducted by trained data collectors and supervisors using a pretested questionnaire. A census of each cluster will be conducted electronically using tablets. Twenty households from each cluster will be selected randomly for the household survey. For the selected households, information collected will include socio-demographic characteristics, knowledge about malaria transmission and prevention, ownership, and utilization of LLIN, coverage of IRS, and risk factors for malaria infection and access to other malaria control interventions. In addition, all members of the selected households will be tested for malaria to estimate the malaria prevalence in the kebele and those turned positive will be treated according to the national treatment guideline. DBS samples will be collected for further testing.

#### Intervention data

In the control arm data related to important indicators for the study will be collected using a standardize form by the woreda surveillance officer. The information will be collected from the standard HP/HC registration books and reporting forms. In the RCD and tMDA arm, data will be collected using a standardized form prepared for the study. The information will be collected from the standard HP/HC registration books and from the study participants.

### Plans to promote participant retention and complete follow-up {18b}

Study participants will remain in the study until they finish the intervention treatment. Participants are encouraged to visit the study sites for follow-ups and reminded via phone call or home visit.

### Data management {19}

Data collection will be performed using a combination of manual data entry and electronic forms. Data collected in electronic format will be sent in real-time to a central server. Daily feedback will be given to the field team by the data managers. Data collected using manual data entry from all arms will be managed, double entered, cleaned, and verified at the central level. At the end of the study, the cleaned dataset will be shared with the investigators for analysis.

### Confidentiality {27}

All information regarding the participants will remain confidential to the extent allowed by law. Unique identifiers will only be used for data entry and will not be linked to any personal identifiers other than household GPS coordinates. GPS coordinates will be offset by a distance, to allow for standardized spatial analyses, but not the exact location of the household.

All case report forms will be kept in a secured location at HPs and transferred to the Addis Continental Institute of Public Health office. Electronic data will be password protected with access limited to authorized study staff. Unique identifiers will be used for the computer-based data entry and blood samples and will not be linked to personal identifiers. Publications will contain only aggregated data; no identifying information will be included.

### Plans for collection, laboratory evaluation, and storage of biological specimens for genetic or molecular analysis in this trial/future use {33}

The study will collect DBS to measure malaria antigen, antibody, and molecular (asexual and sexual) prevalence. A finger prick blood sample (~ 0.3 ml) will be collected from consenting study participants. No human genetic studies will be performed on stored samples. The samples obtained from the study participants will be de-identified when stored for 5 years and will be destroyed thereafter.

## Statistical methods

### Statistical methods for primary and secondary outcomes {20a}

#### Measures of association

Association of different factors such as travel history, education status, occupation, and sex, with the outcomes of interest, will be analyzed using univariate and bivariate tests; all analyses will factor in survey design, weights, and clustering as needed.

Baseline survey and routine surveillance data will be used to determine the prevalence and API for each arm, respectively, at the start of the study. Data from surveys and routine surveillance will also be used to measure the rate of change in the intervention versus control group, at baseline versus endline time points. Interrupted time series regression models will be used to measure the relative percentage change in API, assuming a 95% confidence interval. The proportion of individuals who adhere to the treatment will be determined by self-report, counting pills, and calculating the number of individuals completing the full, partial, or no dose of medication in the intervention and control groups. The proportion of malaria infections that would be missed by screening will be estimated by counting the number missed by the diagnostic method, compared to a more sensitive diagnostic tool such as antigen-based multiplex PCR. The sensitivity and specificity of RDT(s) against more sensitive diagnostic tool(s) will also be calculated. A difference in difference approach with logistic regression will be used to assess the impact on malaria parasite prevalence between arms using both baseline and endline survey data.

#### Cost analysis

Costs will be calculated using an ingredients approach that involves enumerating both the quantity of specific inputs (e.g., hours spent, cost of treatment, number of RDTs used) and the time spent during the intervention. Existing infrastructure and recurrent salary inputs that would be present in the absence of the intervention will not be included in the cost analysis. Although the emphasis of the cost analysis is on determining the cost of RCD and tMDA alone, the cost-effectiveness will then be measured through malaria cases averted as measured through the difference in API in all study kebeles

### Interim analyses {21b}

There will be no planned interim analyses.

### Methods for additional analyses (e.g., subgroup analyses) {20b}

At this time, there is no planned additional subgroup or adjusted analyses.

### Methods in analysis to handle protocol non-adherence and any statistical methods to handle missing data {20c}

The study will use several strategies to ensure that participants adhere to the assigned intervention, including tracking lost to follow-up participants. However, we recognize that some non-adherence is unavoidable. Therefore, the result of the study will be analyzed per protocol. The number of participants with missing information will be clearly stated and the missing data will be mentioned.

### Plans to give access to the full protocol, participant-level data and statistical code {31c}

The manuscript is the full protocol. Anyone interested in the consent forms, de-identified participant-level data, or any future statistical code can contact the authors.

## Oversight and monitoring

### Composition of the coordinating center and trial steering committee {5d}

At each study sites the HEWs and surveillance assistants are responsible for the trial day-to-day activities and ensure the trial is in accordance with the study protocol. The zonal level study coordinator will follow the day-to-day activities and motivate the site study team to recruit all eligible participants, assist with all trial-related questions. A weekly report will be given to the central study coordinator. A monthly site supervision will be conducted to ensure the sites are collecting high-quality data and DBS, and to follow the trial is in accordance with the good clinical practice guidelines. A group of community representatives will be identified after the consultative meeting to serve as a community advisory and engagement committee. Community engagement training will be provided for the committee members and HEWs. Community engagement activities will include house-to-house communication through community advisory and engagement committees. The information provided will focus on why the specific community is in that arm (tMDA, RCD, or control) and, why, when, and how RCD or tMDA will be carried out. All investigators will meet at a regular interval to monitor the progress and review the relevant information.

### Composition of the data monitoring committee, its role, and reporting structure {21a}

An independent data and safety monitoring board (DSMB), composed of a multidisciplinary group of experts will be established. The DSMB will assess trial progress quarterly and report the ongoing scientific and ethical integrity of the study to investigators and responsible agencies. The DSMB will ensure that the study is conducted and the data are handled in accordance with the provisions of the research protocol.

### Adverse event reporting and harms {22}

All SAEs and unanticipated problems, regardless of whether there is sufficient evidence to establish causality with the study drug, will be reported to the PI. The PI then will report to all relevant IRBs within 48 hours of the event occurring. Representatives from the study team, including at least one member from each participating institution, will review the event to establish definite causality, probable causality, possible causality or no causality with the study drug or procedures for administering the drug. In the event of a drug-related SAE, Armauer Hansen Research Institute (AHRI) will convene a meeting within a week of the event with the representatives from the study team, the MOH, and Addis Continental Institute of Public Health (ACIPH), to review the case and take any remediating action deemed necessary.

### Frequency and plans for auditing trial conduct {23}

The research team will have a regular meetings to ensure enrollment, recruitment, and follow-ups are in accordance with the study protocol. The study is subjected to submit annual report and renewal of the license from the institutional and national ethical review boards.

### Plans for communicating important protocol amendments to relevant parties (e.g., trial participants, ethical committees) {25}

Any amendments will be submitted to the institutional and national ethical review boards prior to implementation. The protocol amendments will be communicated to relevant authorities. epidemiology and biostatistics

### Dissemination plans {31a}

The trial results will be disseminated to all concerning bodies by organizing a dissemination workshop. In addition, the results will be disseminated through academic conferences and published in peer-reviewed journals.

## Discussion

This study is a three-arm, cluster-randomized, open-label, controlled intervention trial conducted over 2 years, which has aim of evaluating the impact of tMDA and RCD within 100 m of the index case compared to standard malaria control intervention in reducing malaria incidence in low transmission settings of Ethiopia. A team of researchers from MoH, AHRI, ACIPH, Tulane University, PMI, and other relevant partners from in-country and abroad are involved in this evaluation. The outcome of this study will contribute to the impact and further targeting of Ethiopian MoH malaria elimination interventions strategies.

The baseline survey was conducted in January 2020 as scheduled. Although our timeline was to initiate the interventions, immediately after the baseline assessment, we could not make that happen because of the COVID-19 crisis in the country and the limitation of movement. Therefore, the intervention started in November 2020. We acknowledge the delay to start the intervention as planned shortened the planned intervention time.

## Trial status

The study is ongoing. The baseline survey was conducted form January to February 2020. The intervention started in November 2020. There was a delay due to the COVID-19 pandemic. Participant recruitment is ongoing and expected to end in January 2022. Endline survey is scheduled for January to February 2022.

## Abbreviations

ACIPH: Addis Continental Institute of Public Health
API: Annual parasite incidence
AHRI: Armauer Hansen Research Institute
AL: Artemether-Lumefantrine
CQ: Chloroquine
DSMB: Data and Safety Monitoring Board
DBS: Dried blood spot
G6PD: Glucose-6-phosphate dehydrogenase
HEWs: Health extension workers
HC: Health center
HP: Health post
Hgb: Hemoglobin
IRS: Indoor residual spraying
IRB: Institutional Review Board
LLINs: Long-lasting insecticidal nets
MOH: Ministry of Health
PQ: Primaquine
RCD: Reactive case detection
RDT: Rapid diagnostic test
SAE: Serious adverse event
tMDA: Targeted mass drug administration
